# Complete mitochondrial genome of the *Oncorhynchus keta* (Walbaum, 1792) and *Oncorhynchus masou masou* (Brevoort, 1856)

**DOI:** 10.1080/23802359.2017.1298412

**Published:** 2017-04-13

**Authors:** Peilun Li, Wei Liu, Jilong Wang, Fujiang Tang

**Affiliations:** Heilongjiang River Fisheries Research Institute, Chinese Academy of Fishery Sciences, Harbin, China

**Keywords:** Complete mitochondrial genome, *Oncorhynchus keta*, *Oncorhynchus masou masou*

## Abstract

In this study, we sequenced and determined the complete mitochondrial genome of *Oncorhynchus keta* and *Oncorhynchus masou masou*, which are the economically important fish species of China. They are circular molecule of 16,656 and 16,704 bp in size, containing 13 protein-coding genes, 22 transfer RNAs, 2 ribosomal RNAs and a displacement loop region (D-loop). *Oncorhynchus keta* mitochondrial genome consists of A: 27.80%, T: 26.21%, G: 16.98%, C: 29.01%. *Oncorhynchus masou masou* mitochondrial genome consists of A: 28.20%, T: 26.82%, G: 16.52%, C: 28.46%. The total length of the 13 protein-coding genes was 11,420 and 11,415 bp.

*Oncorhynchus keta* and *Oncorhynchus masou masou* are belong to *Salmonoidea* family, which are the economically important fish species in China. *Oncorhynchus keta* is widely distributed in North pacific and its coastal tributary, which is the typical anadromous fish (Liu et al. 2013). Compared to *O. keta*, the distritution range of *O. masou masou* is relatively centralized in Northwest Pacific, which occurs in two life history forms: anadromous and landlocked (Xie 2007). In China, the *O. keta* is distributed in Amur, Wusuli, Suifen Current and Tumen Rivers, and the *O. masou masou* is distributed in Tumen River and Suifen Current. At present, there are few reports about *O. keta* and *O. masou masou* from rivers in northeast of China (Chen 2005; Liu et al. 2013; Wang et al. 2013, 2015). Mitochondrial DNA information provided the basis for the studies in population genetic differentiation. In this study, the complete sequence of the mitochondrial genome of *O. keta* and *O. masou masou* (GeneBank accession number are KY320492 and KY320493) was discovered, which will be suitable for biogeographical and population genetic analyses of the salmons.

*Oncorhynchus keta* was collected from Wusuli River, which is a natural boundary between China and Russia. *Oncorhynchus masou masou* was collected in the middle reaches of Suifen River. The total genomic DNA was extracted with the traditional phenol-chloroform method (Taggart et al. 1992). Twenty-eight and 35 primers were used to amplify the target PCR products as sequencing, it is designed by primers and PCR product was purified by 3.0 Analysis ContigExpess softword (SAS Inc., Cary, NC). They are circular molecule of 16,656 and 16,704 bp in size, containing 13 protein-coding genes, 22 transfer RNAs, 2 ribosomal RNAs and a displacement loop region (D-loop). Most of the genes are encoded on the heavy strand (H-strand), except for the eight tRNA genes (tRNA-Gln, tRNA-Ala, tRNA-Asn, tRNA-Cys, tRNA-Tyr, tRNA-Ser, tRNA-Glu and tRNA-Pro) and one protein-coding gene (ND6). *Oncorhynchus keta* mitochondrial genome consists of A: 27.80%, T: 26.21%, G: 16.98%, C: 29.01%. *Oncorhynchus masou masou* mitochondrial genome consists of A: 28.20%, T: 26.82%, G: 16.52%, C: 28.46%. The total length of the 13 protein-coding genes was 11,420 and 11,415 bp.

The complete mitochondrial genome sequences of 15 other closely related species are downloaded from GenBank, which were used for phylogenetic analysis (Figure 1). Multiple alignments of 17 mitochondrial genomes were aligned using ClustalW. The phylogenetic tree from maximum-likelihood was reconstructed using Mega6.0 (Tamura et al. 2013). To obtain the confident supports, 1000 bootstrap replicates were set for the analysis. Pholygenetis analysis showed that *O. masou masou* shared the same cluster with other *O. masou* subspecies and *O. keta* shared the same cluster with other *Oncorhynchus* subspecies, they are closely relative species but they may diverge differently. We expect that the present result will help to elucidate the taxonomic status of *O. masou masou* and *O. keta*, which contribute to the conservation.

**Figure 1.**
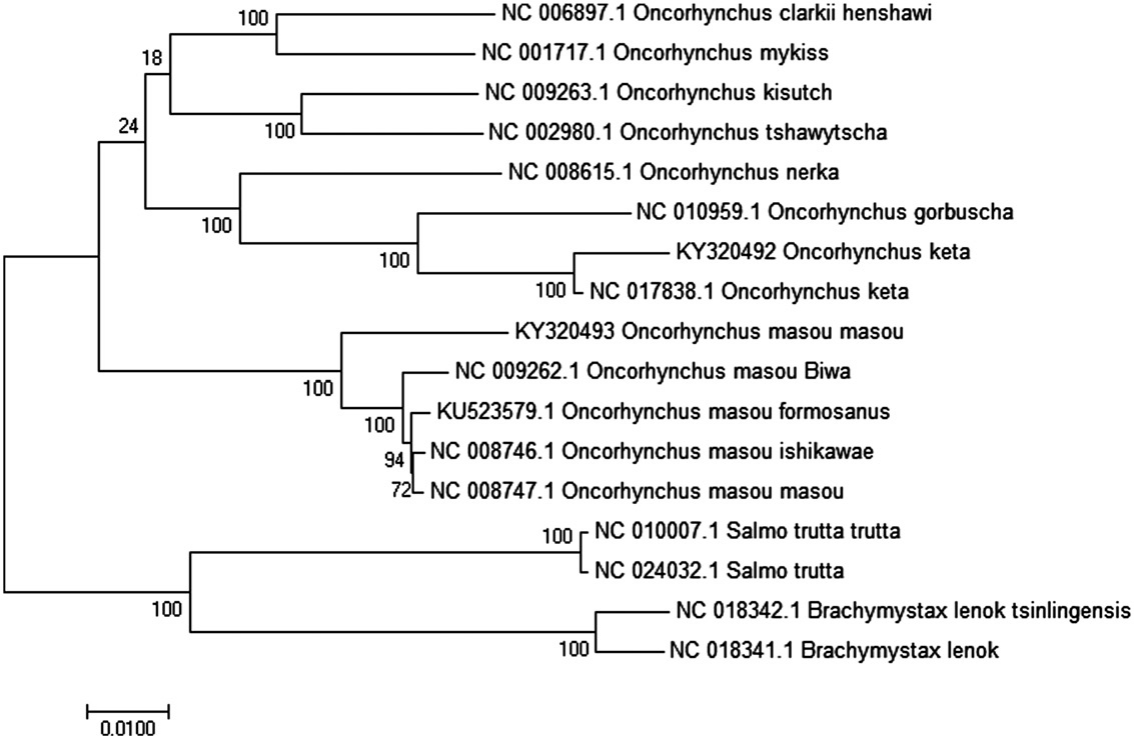
Phylogeny based on the complete mitochondrial genomes by Mega6.0 with 1000 bootstrap replications. The maximum-likehood tree is drawn to scale.

## References

[CIT0005] Chen JP. 2005 Genetic analysis of four wild Chum salmon *Oncorhynchus keta* populations in China based on microsatellite markers. Environ Biol Fish. 73:181–188.

[CIT0001] Liu W, Zhan PR, Wang JL, Tang FJ. 2013 Structure of otoliths dailt growth ring of embryo Chum salmon and environmental mass marking. Acta Hydrobiol Sin. 37:929–937.

[CIT0006] Taggart JB, Hynes RA, Prodoh PA, Ferguson A. 1992 A simplified protocol for routine total DNA isolation from salmonid fishes. J Fish Biol. 40:963–965.

[CIT0007] Tamura K, Stecher G, Peterson D, Filipski A, Kumar S. 2013 MEGA6: molecular evolutionary genetics analysis version 6.0. Mol Biol Evol. 30:2725–2729. 24132122 10.1093/molbev/mst197 PMC3840312

[CIT0002] Wang JL, Liu W, Tang FJ. 2013 Analysis of biological traits of Chum salmon (*Oncorhynchus keta* Walbaum) in the Amur River, China. J Fish Sci China. 20:93–100.

[CIT0003] Wang C, Liu W, Zhan PR, Wang JL, Li PL. 2015 Exogenous Sr^2+^ sedimentation on otolith of Chum salmon embrys. Chin J Appl Ecol. 26:3189–3194. 26995930

[CIT0004] Xie YH. 2007 Freshwater Fishes in Northeast Region of China. Shenyang, China: Liaoning Science and Technology Publishing House, pp. 296–300.

